# High sugar intake via the renin-angiotensin system blunts the baroreceptor reflex in adult rats that were perinatally depleted of taurine

**DOI:** 10.1186/1423-0127-17-S1-S30

**Published:** 2010-08-24

**Authors:** Atcharaporn Thaeomor, J Michael Wyss, Dusit Jirakulsomchok, Sanya Roysommuti

**Affiliations:** 1 Department of Physiology Faculty of Medicine, Khon Kaen University, Khon Kaen 40002, Thailand; 2 Department of Cell Biology, University of Alabama at Birmingham, AL 35294, USA

## Abstract

Perinatal taurine depletion leads to several physiological impairments in adult life, in part, due to taurine’s effects on the renin-angiotensin system, a crucial regulator of growth and differentiation during early life.  The present study tests the hypothesis that perinatal taurine depletion predisposes adult female rats to impaired baroreceptor control of arterial pressure by altering the renin-angiotensin system.  Female Sprague Dawley (SD) rats were fed normal rat chow and from conception to weaning drank 3% beta-alanine in water (taurine depletion, TD) or water alone (Control, C).  Female offspring ate a normal rat chow and drank water with (G) or without (W) 5% glucose throughout the experiment.  To test the possible role of the renin-angiotensin system, 50% of the rats received captopril (an angiotensin converting enzyme inhibitor, 400 mg/L) from 7 days before parameter measurements until the end of experiment.  At 7-8 weeks of age, arterial pressure, heart rate, baroreflex control of heart rate and renal nerve activity were studied in either conscious, freely moving or anesthetized rats.  Perinatal taurine depletion did not alter resting mean arterial pressure or heart rate in the adult female offspring that received either high or normal sugar intake.  Captopril treatment slightly decreased mean arterial pressure but not heart rate in all groups.  Compared to controls, only the TDG rats displayed blunted baroreflex responses.  Captopril treatment normalized baroreflex sensitivity in TDG.  The present data indicate that in perinatal taurine depleted female rats, the renin-angiotensin system underlines the ability of high sugar intake to blunt baroreceptor responses.

## Introduction

Taurine is present at a high concentration in many organs including brain, heart, kidneys, and reproductive organs.  Taurine content in these organs is highest during the perinatal period, and it modestly declines with advancing age [1].  Several lines of evidence indicate that in addition to other aspects of the perinatal environment (e.g., nutrition and hormones), taurine contributes to programming adult function and diseases susceptibility, especially in relation to the cardiovascular system [2].  Poor nutrition in early life can lead to obesity, diabetes mellitus, hypertension and coronary heart diseases in adults [3], and via epigenetic mechanisms, these can transfer to the next generation.  In addition, perinatal inhibition of the renin-angiotensin system impairs renal function [4,5] and induces salt-sensitive hypertension in normotensive animals but prevents hypertension in spontaneously hypertensive rats [6,7].

Taurine supplementation either during perinatal period or during young adult life prevents hypertension in adult spontaneously hypertensive rats, partly by its antioxidant action [8].  Exposure to excess taurine in perinatal life influences growth and autonomic nervous system control of arterial pressure in adult male rats [9,10].  Renal hemodynamics are sensitive to perinatal taurine action [11], as demonstrated by the finding that taurine transporter knockout mice display several abnormalities in renal structure and function in adult life [12,13].  In addition, perinatal taurine depletion heightens sugar-induced hypertension in the adult male offspring [10], and this effect impairs renal function prior to the appearance of hypertension and diabetes mellitus [9,14].  Renin-angiotensin system overactivity underlines this phenomenon.

The present study tests the hypothesis that perinatal taurine depletion via renin-angiotensin mechanisms impairs baroreceptor reflex control of arterial pressure in adult female rats.  Hyperinsulinemia and insulin resistance are also investigated.

## Materials and methods

### Animal preparation

Sprague Dawley (SD) rats were bred from the animal unit of Faculty of Medicine, Khon Kaen University and maintained at constant humidity (60 ± 5%), temperature (24 ± 1 ^o^C), and light cycle (0600-1800 h).  Female SD rats were fed normal rat chow and drank 3% beta-alanine in water (taurine depletion, TD) or water alone (Control, C) from conception to weaning.  Female offspring were then fed the normal rat chow with either 5% glucose in tap water (G) or tap water alone (W) throughout the experiment.  To inhibit the renin-angiotensin system, captopril (an angiotensin converting enzyme inhibitor, 400 mg/L) was administered in drinking water of 50% of the rats in each group from 7 days before initial testing until the end of experiment.  At 7-8 weeks of age, arterial pressure and heart rate and baroreflex control of heart rate and renal nerve activity were tested in either conscious, freely moving or anesthetized (thiopental sodium, 50 mg/kg, i.p.) rats.  All experimental procedures were preapproved by the Khon Kaen University Animal Care and Use Committee and were conducted in accordance with the National Institutes of Health guidelines.

### Experimental protocol

At the time of study, all female rats were anesthetized by thiopental sodium and then were implanted with femoral arterial and venous catheters.  Two or three days later, after an overnight fast, arterial blood samples (1.0 ml each) were taken from conscious rats and analyzed for Na, K, hematocrit, blood urea nitrogen, creatinine, insulin, and fasting blood sugar determinations.  Blood volumes were immediately replaced with equal volumes of donor blood from rats of same treatment.  Twenty-four hours later, arterial pressure was continuously recorded (BIOPAC, Goleta, CA) in conscious rats before and during infusion of phenylephrine (PE; to increase arterial pressure) or sodium nitroprusside (SNP; to decrease arterial pressure).  One-day later, female rats were anesthetized with thiopental sodium and tracheostomized, and arterial pulse was recorded continuously for assessment of baroreflex control of renal nerve activity.  Body temperature was servo-control at 37±0.5°C by a rectal probe connected to a temperature regulator controlling an overhead heating lamp.  At the end of experiment, all animals were sacrificed and kidney and heart weights were then measured.

### Data analyses

Single unit recording of renal sympathetic nerve activity was assessed by inserting a stainless steel electrode (12 MΩ, 0.01 Taper, A-M system, FL, USA) through surgically opened abdomen.  The electrode holder was connected to a DAM-80 amplifier (1,000x, 500-1,000 Hz band pass filter; WPI, USA) and the BIOPAC MP 100 system (5,000 samples per second).  Changes in renal nerve activity and heart rate were compared to mean arterial pressure following sodium nitroprusside or phenylephrine infusion to determine baroreceptor reflex sensitivity control of renal nerve activity (BSRA-S or BSRA-P) and heart rate (BSHR-S or BSHR-P).  All offline data analyses were performed by the Acknowledge Software (BIOPAC).

All blood chemistry parameters were analyzed by the Khon Kaen University Hospital laboratory, with a blind control procedure.  All data were expressed as mean ± SEM.  Statistical comparisons among groups (p < 0.05) were performed by using one-way ANOVA and Duncan’s Multi-Range tests (StatMost ver. 3.6 software, DataMost, USA).

## Results

At 7-8 weeks of age, body, kidney, heart, kidney to body, and heart to body weights were not significantly different among the experimental groups (Table 1).  Plasma sodium, plasma potassium, plasma creatinine, blood urea nitrogen, fasting blood sugar, and hematocrit were not significantly different among the groups (Table 2).  Compared to CW, plasma insulin levels in CG groups were significantly higher, and captopril treatment eliminated this difference.  Plasma insulin levels were not significantly different between TDW and TDG, but they were significantly higher than those in CW (about 2 time) and CG groups.  In contrast to CG, captopril treatment markedly increased plasma insulin in TDW and TDG and moderately increased it in CW.

**Table 1:** Body (BW), kidney (KW), and heart (HW) weights in experimental groups

Treatment	BW (g)	HW (g)	KW (g)	HW/BW (%)	KW/BW (%)
**CW (n=7)**	183 ± 2	0.66 ± 0.01	0.69 ± 0.01	0.36 ± 0.01	0.38 ± 0.01
**CW+C (n=7)**	183 ± 3	0.70 ± 0.02	0.78 ± 0.02	0.38 ± 0.01	0.43 ± 0.01
**CG (n=7)**	182 ± 3	0.73 ± 0.03	0.78 ± 0.04	0.40 ± 0.02	0.43 ± 0.02
**CG+C (n=7)**	188 ± 2	0.84 ± 0.02	0.93 ± 0.01	0.45 ± 0.01	0.50 ± 0.01
**TDW (n=7)**	185 ± 3	0.72 ± 0.02	0.75 ± 0.02	0.39 ± 0.01	0.41 ± 0.01
**TDW+C (n=7)**	181± 4	0.71 ± 0.03	0.76 ± 0.02	0.39 ± 0.01	0.42 ± 0.00
**TDG (n=7)**	187 ± 3	0.81 ± 0.03	0.85 ± 0.03	0.43 ± 0.01	0.46 ± 0.02
**TDG+C (n=7)**	187 ± 3	0.79 ± 0.04	0.88 ± 0.04	0.42 ± 0.02	0.47 ± 0.01

Data were mean ± SEM.  No significant difference among groups was noted (CW, control with water intake alone; CW+C, CW plus captopril treatment; CG, control with high sugar intake; CG+C, CG plus captopril treatment; TDW, perinatal taurine depletion with water intake alone ; TDW+C, TDW plus captopril treatment; TDG, perinatal taurine depletion with high sugar intake; TDG+C, TDG plus captopril treatment; TSW, perinatal taurine supplementation with water intake alone; TSW+C, TSW plus captopril treatment; TSG, perinatal taurine supplementation with high sugar intake; TSG+C, TSG plus captopril treatment).

**Table 2:** General blood chemistry in experimental groups

Treatment	Na (mEq/l)	K (mEq/l)	BUN (mg/dl)	Cr (mg/dl)	FBS (mg/dl)	Insulin (pmol/l)	Hct (%)
**CW (n=7)**	142.9 ± 2.9	3.5 ± 0.2	20.6 ± 1.3	0.51 ± 0.03	102.4 ± 3.1	14.8 ± 1.4	48.1 ± 1.2
**CW+C (n=7)**	140.2 ± 2.8	3.9 ± 0.2	21.5 ± 1.7	0.55 ± 0.02	92 ± 3.5	26.0 ± 3.3 *	45.0 ± 2.6
**CG (n=7)**	141.5 ± 1.8	3.7 ± 0.1	22.8 ± 1.8	0.53 ± 0.03	103.2 ± 3.8	18.2 ± 0.9 *	44.8 ± 2.1
**CG+C (n=7)**	144.8 ± 2.9	3.7 ± 0.2	22.4 ± 1.1	0.54 ± 0.03	94.2 ± 2.1	15.7 ± 1.5^ £^	47.8 ± 1.1
**TDW (n=7)**	137.8 ± 2.5	3.6 ± 0.2	20.8 ± 1.8	0.53 ± 0.03	93.8 ± 6.3	26.8 ± 1.5 *	47.1 ± 1.6
**TDW+C (n=7)**	144.1 ± 2.7	3.9 ± 0.3	22.0 ± 1.6	0.56± 0.02	94.1 ± 2.9	44.0 ± 2.6 *^,€^	48.8 ± 1.7
**TDG (n=7)**	136.3 ± 2.9	3.5 ± 0.2	21.9 ± 1.2	0.56 ± 0.02	92 ± 4.7	27.4 ± 3.3 *	44.1 ± 2.3
**TDG+C (n=7)**	149.8 ± 2.4	3.6 ± 0.1	23.0 ± 1.9	0.55 ± 0.03	97.5± 1.5	73.8 ± 7.9 *^,€,£^	47 ± 1.9

Data were mean ± SEM (* P<0.05 compared to CW, ^€^ to corresponding groups without captopril, ^£^ to corresponding groups with captopril;  Na, plasma sodium, K, plasma potassium, BUN, blood urea nitrogen, Cr, plasma creatinine, FBS, fasting blood sugar, Hct, hematocrit). For treatment abbreviations, see Table 1

**Figure 1:**
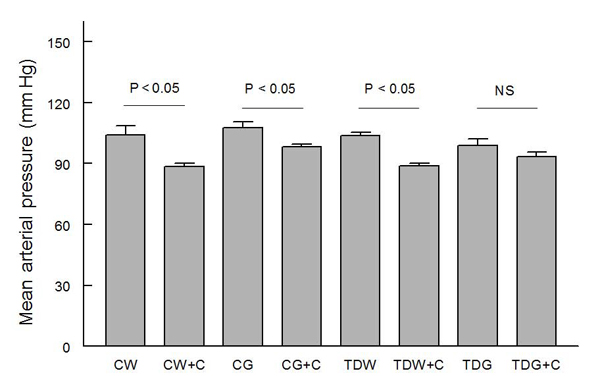
**Comparison of resting mean arterial pressure among animal groups with different treatments** (CW, control with water intake alone; CW+C, CW plus captopril treatment; CG, control with high sugar intake; CG+C, CG plus captopril treatment; TDW, perinatal taurine depletion with water intake alone ; TDW+C, TDW plus captopril treatment; TDG, perinatal taurine depletion with high sugar intake; TDG+C, TDG plus captopril treatment)

**Figure 2:**
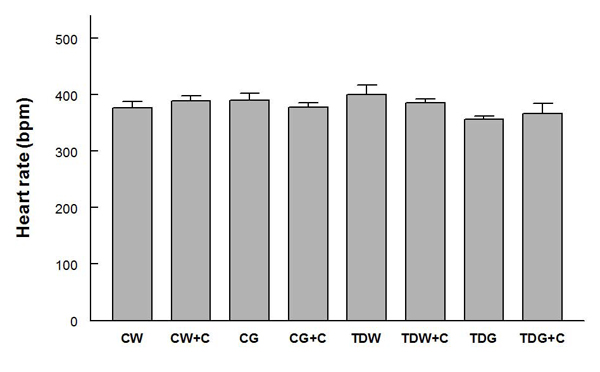
**Comparison of resting heart rate among animal groups with different treatments** (for treatment abbreviations, see Figure 1)

**Figure 3:**
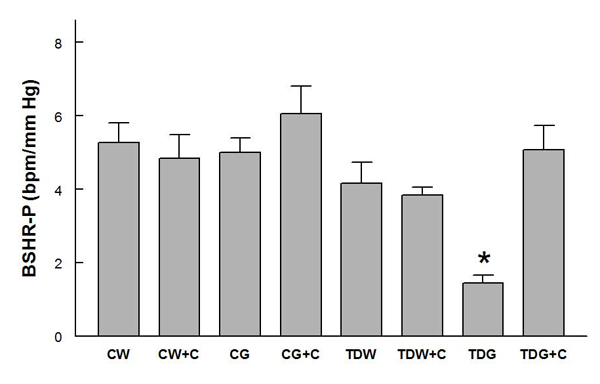
**Baroreflex sensitivity control of heart rate tested by phenylephrine infusion (BSHR-P) in conscious rats** (* P < 0.05, compared to all other groups; for treatment abbreviations, see Figure 1)

**Figure 4:**
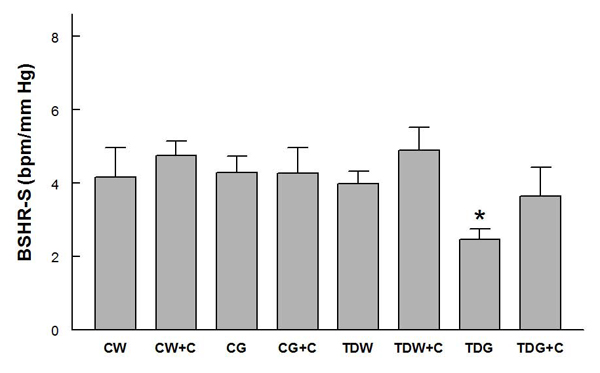
**Baroreflex sensitivity control of heart rate tested by sodium nitroprusside infusion (BSHR-S) in conscious rats** (* P < 0.05, compared to all other groups; for treatment abbreviations, see Figure 1)

Mean arterial pressures were not significantly different among the groups, and they were significantly decreased by captopril treatment in all groups except TDG (Fig. 1).  Heart rates were not significantly different among groups (Fig. 2).  PE-induced baroreflex control of heart rate was not significantly different among control groups.  In the TD groups, heart rate baroreflex was significantly impaired by glucose treatment, but this was returned to control levels by captopril treatment (Fig. 3).  Captopril treatment did not alter these responses in the other groups.  A similar pattern was observed in responses to SNP (Fig. 4).

**Figure 5:**
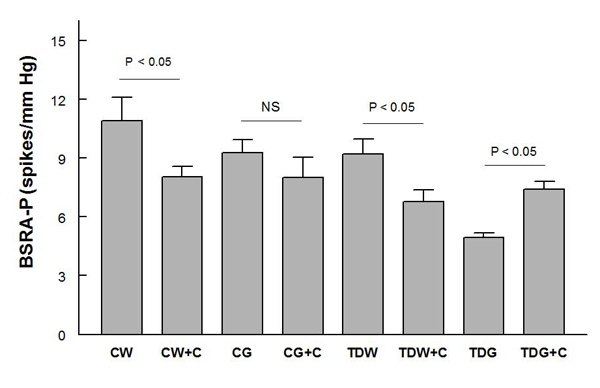
**Baroreflex sensitivity control of renal sympathetic nerve activity tested by phenylephrine infusion (BSRA-P) in anesthetized rats** (* P < 0.05; for treatment abbreviations, see Figure 1)

**Figure 6:**
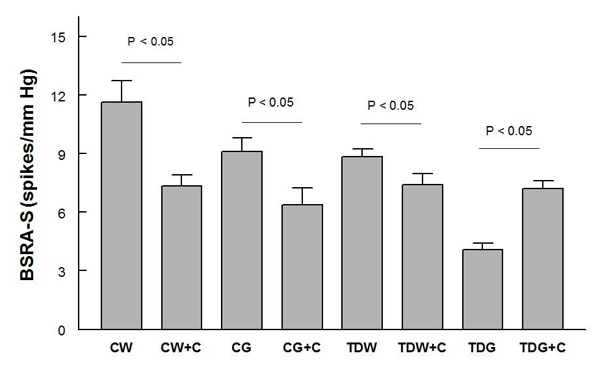
**Baroreflex sensitivity control of renal sympathetic nerve activity tested by sodium nitroprusside infusion (BSRA-S) in anesthetized rats** (* P < 0.05; for treatment abbreviations, see Figure 1)

Baroreflex sensitivity control of renal sympathetic nerve activity following either PE (Fig. 5) or SNP (Fig. 6) decreased significantly only in TDG, and this was returned to control levels by captopril treatment.  Interestingly, captopril treatment slightly decreased this measure in the other three groups (Fig. 6).

## Discussion

Renin-angiotensin mechanisms underlie sugar-induced hypertension in many animal models [15].  High sugar intake (similar to that in the present study) induces renal dysregulation prior to its effects on hypertension and diabetes mellitus, and these changes are related to renin-angiotensin system overactivity [16].  The present data indicate that the renin-angiotensin system contributes to the blunted baroreflex control of arterial pressure in perinatal taurine-depleted adult female rats on a high sugar diet.  In perinatal taurine-depleted adult male rats [10], high sugar intake increases sympathetic nerve activity and blunts baroreflex sensitivity, but the interplay of the renin-angiotensin system in this male model has not been tested.

The sympathetic nerves stimulate renin release from juxtraglomerular cells of the kidneys.  Further, angiotensin II can activate the sympathetic nerves and norepinephrine release in the periphery and act in the anterior hypothalamus and rostral ventrolateral medulla [17,18].  Thus, the linkage between sympathetic nervous system, norepinephrine release, and the renin-angiotensin system can contribute to the genesis of many disorders via complex interactions.  In spontaneously hypertensive rats, lifetime treatment with captopril prevents hypertension in adult life [7], but this treatment does not block dietary salt-sensitive hypertension in these animals.  The salt-sensitive hypertension is related to sympathetic nervous system overactivity [6].  In sugar-induced hypertension, both the sympathetic nervous system and the renin-angiotensin system appear to play a major role.  While the renin-angiotensin system may be the primary contributor to renal salt and water retention during the early phase of hypertension, both the sympathetic nervous and renin-angiotensin systems contribute importantly to sustained hypertension [15,19,20].

Sugar-induced hypertension involves not only the sympathetic nervous system and the renin-angiotensin overactivity, but also hyperinsulinemia and insulin resistance [21].  The present findings confirm our previous reports that high sugar intake does not induce hyperglycemia and glucose intolerance in either control or perinatal taurine depleted female or male rats [9], but the present data indicate that although all animals displayed euglycemia, the insulin sensitivity of the groups were different (as estimated by the ratio of plasma glucose to insulin).  During normal taurine exposure, high sugar intake (from weaning onward) slightly increases the plasma insulin concentration, but this is restored to control levels after captopril treatment.  The finding that angiotensin II contributes to mild insulin resistance is supported by previous experiments [21].  Angiotensin II acts on insulin’s target cells and alters post-receptor mechanisms to produce insulin resistance [22].  The insulin resistance may subsequently limit insulin clearance, resulting in increased plasma insulin.  Renal metabolism and elimination of insulin is receptor-mediated [23,24].  Further, the renin-angiotensin system inhibits pancreatic, glucose-stimulated insulin secretion and biosynthesis and activates superoxide production that can induce pancreatic damage [25,26].  Thus, inhibition of the local renin-angiotensin system likely contributes to the insulin effects in the taurine depleted, sugar fed female rats in this study.

Perinatal taurine depletion increased plasma insulin concentration, but this was not further increased by high sugar intake.  In contrast, captopril treatment greatly increased plasma insulin levels in taurine-depleted rats.  The high sugar intake exacerbated this increase.  In addition, fasting blood glucose was not significantly different among groups, suggesting that perinatal taurine depletion induces insulin resistance in female rats.  It is well known that inhibition of the renin-angiotensin system increases insulin sensitivity and insulin secretion in humans and animals [22,27].  A marked rise in insulin resistance in TDG+C more than TDW+C (vs. normalization in CG+C) rats indicates an abnormal insulin/angiotensin II relationship in these animals that is exacerbated by the high sugar intake.  An abnormal insulin feedback inhibition may also contribute.

Inhibition of the renin-angiotensin system restored baroreflex sensitivity of the perinatal taurine-depleted rats to that of control groups, despite their hyperinsulinemia and insulin resistance.  This suggests that renin-angiotensin system overactivity primarily underlies this baroreflex depression.  However, insulin enhances baroreflex sensitivity via the central nervous system [22,28,29].  TDG and TDW displayed similar decreased insulin sensitivity, but the baroreflex sensitivity was blunted only in TDG rats, suggesting that that blunted baroreflex is not due to insulin resistance alone. Whether a rise in plasma insulin level despite insulin resistance normalizes the baroreflex sensitivity in TDG+C rats has to be further clarified.

In adult animals, taurine supplementation improves insulin resistance and hypertension in sugar-induced hypertension by inhibition of angiotensin II  and its oxidative and other actions [22,30,31].  It also reduces the cardiac hypertrophic effect of angiotensin II in animals [32,33].  In contrast, taurine deficiency in mature animals accelerates many adverse actions of angiotensin II on the heart, blood vessels, and kidney function [33,34].  In the kidney, taurine increases water and sodium excretion while angiotensin II acts in an opposite direction.  In the medulla, angiotensin II injection increases taurine secretion [35].  Moreover, taurine stimulates, while angiotensin II inhibits, islet insulin secretion [36-38].  Together, these findings indicate that taurine and the renin-angiotensin system share many physiological roles in human bodies.  The renin-angiotensin system is crucial for growth and development at perinatal life.  Its deficiency produces organ damage and abnormalities in adults, especially related to the kidney [4,5] and the nervous system [39].  Similarly, sufficient taurine exposure during a perinatal period leads to normal adult organ function [1].  The present data confirm these relationships.  Perinatal taurine depletion may increase the renin-angiotensin system activity at early life and this change may lead to permanent effects that cannot be reversed by later taurine availability, i.e., the programming of renin-angiotensin system function in mature life is likely dictated by the perinatal exposure.

## Conclusion

Perinatal taurine depletion does not alter baroreflex sensitivity but induces hyperinsulinemia and insulin resistance.  High sugar intake blunts baroreflex function but does not modify the hyperinsulinemia or insulin resistance. When treated with captopril, the blunted baroreflex is completely normalized, but hyperinsulinemia and insulin resistance is exacerbated.  Thus, the present data indicate that in perinatal taurine depleted adult, female rats, high sugar intake blunts baroreceptor reflexes, at least in part, via renin-angiotensin system overactivity, but insulin resistance does not appear to contribute to the blunted baroreflex.

## List of abbreviations used

CW: control with water intake alone; CW+C: CW plus captopril treatment; CG: control with high sugar intake; CG+C: CG plus captopril treatment; TDW: perinatal taurine depletion with water intake alone; TDW+C: TDW plus captopril treatment; TDG: perinatal taurine depletion with high sugar intake; TDG+C: TDG plus captopril treatment; BW: body weight; HW: heart weight; KW: kidney weight; SD: Sprague Dawley; i.p.: intraperitoneal; PE: phenylephrine; SNP: sodium nitroprusside; BSRA-S or BSRA-P: baroreceptor reflex sensitivity control of renal nerve activity tested by SNP or PE; BSHR-S or BSHR-P: baroreceptor reflex sensitivity control of heart rate tested by SNP or PE; SEM: standard error of means; BUN: blood urea nitrogen; FBS: fasting blood sugar; Hct: hematocrit; K: plasma potassium, Na, plasma sodium; Cr: plasma creatinine

## Competing interests

The authors declare that they have no competing interests.

## Authors’ contributions

1. Atcharaporn Thaeomor: research proposal preparation, data collection and analysis, article preparation

2. J. Michael Wyss: research consult, article preparation

3. Dusit Jirakulsomchok: research consult, article preparation

4. Sanya Roysommuti: research proposal design, data analysis, article preparation, correspondence
